# Supplementary material for: Artificial intelligence in peer review: How can evolutionary computation support journal editors?
Source: PLoS One. 2017 Sep 20;12(9):e0184711. doi: 10.1371/journal.pone.0184711 (PMC5607159; doi:10.1371/journal.pone.0184711)

Table S1. Distribution of the length of the review process (single review thread; process ended with a review) in JSCS.

Days	Freq.	Days	Freq.	Days	Freq.	Days	Freq.
0	1	11	1	22	4	33	2
1	1	12	4	23	6	34	3
2	5	13	3	24	6	35	3
3	3	14	4	25	3	36	3
4	2	15	4	26	4	38	1
5	2	16	3	27	3	39	3
6	3	17	6	28	6	40	2
7	4	18	3	29	4	41	3
8	4	19	3	30	2	53	1
9	1	20	6	31	2	55	1
10	3	21	4	32	3	68	1

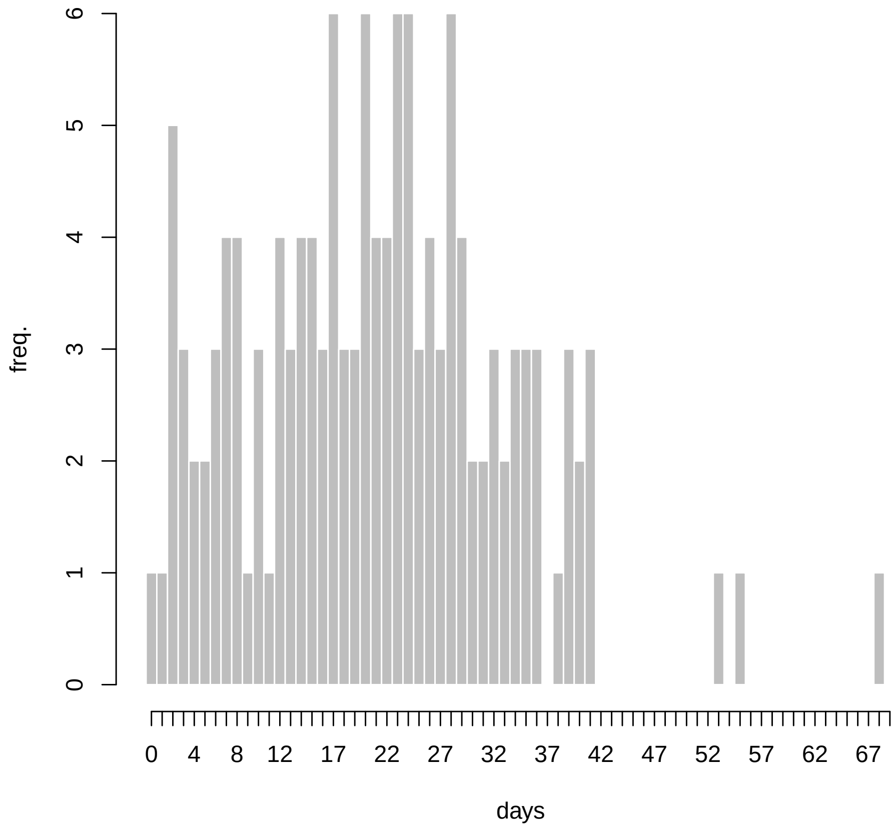

Supplement: S1 Table — (PDF) [file pone.0184711.s001.pdf]
