# Supplementary material for: Artificial intelligence in peer review: How can evolutionary computation support journal editors?
Source: PLoS One. 2017 Sep 20;12(9):e0184711. doi: 10.1371/journal.pone.0184711 (PMC5607159; doi:10.1371/journal.pone.0184711)

Table S2. Distribution of the length of the review process (single review thread; process ended without a review) in JSCS.

Days	Freq.	Days	Freq.
1	5	11	1
2	1	16	4
3	2	17	89
4	1	18	20
8	14	37	1
9	14	40	7
10	15	41	1

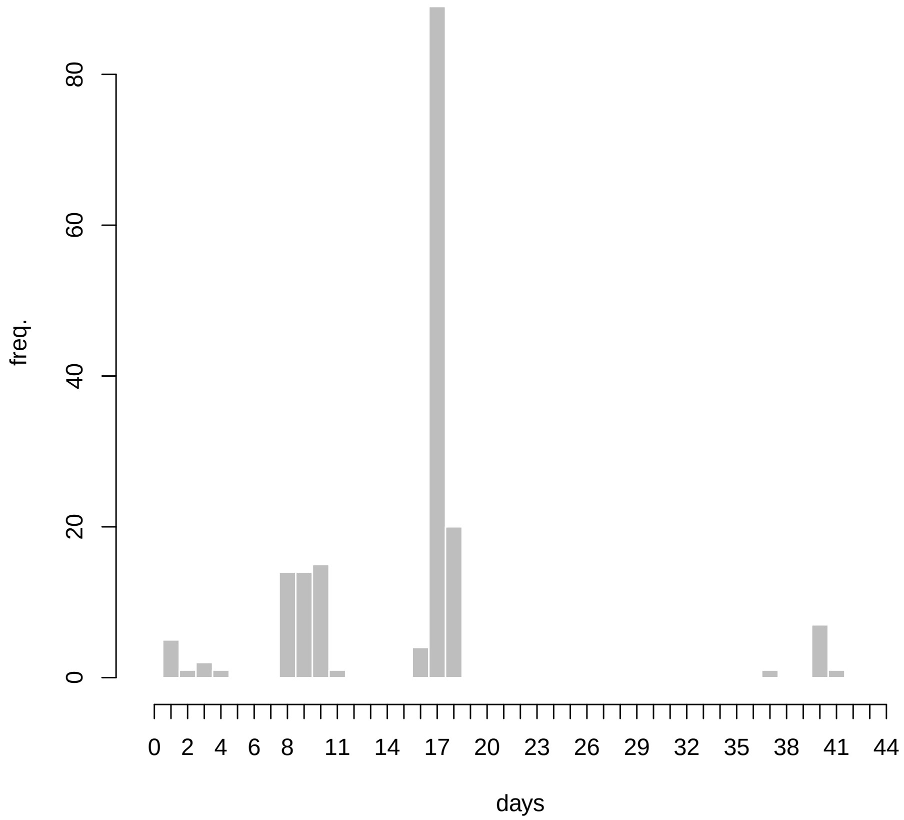

Supplement: S2 Table — (PDF) [file pone.0184711.s002.pdf]
