# Supplementary material for: Artificial intelligence in peer review: How can evolutionary computation support journal editors?
Source: PLoS One. 2017 Sep 20;12(9):e0184711. doi: 10.1371/journal.pone.0184711 (PMC5607159; doi:10.1371/journal.pone.0184711)
Supplement: S1 Algorithm — (PDF) [file pone.0184711.s003.pdf]

**Algorithm S1a:** The algorithm which evaluates the fitness of editorial strategies.

```
Function fitness(editorialStrategy: editorial strategy, minBatch: integer, maxBatch: integer,
minEffective: integer, maxEffective: integer, plannedSimulationRuns: integer, criticalFitness: integer):
    points ← {};                                  // initialise empty list of Point objects
    criticalErrors ← 0;
    foreach batchSize between minBatch and maxBatch do
        point ← (0, 0);                           // initialise Point point to (x = 0, y = 0)
        simulationRuns ← 0;
        repeat
            simulationRuns ← simulationRuns + 1;
            elapsedDays,effectiveReviewers,criticalError ← simulation(batchSize, editorialStrategy);
            if criticalError = true then
                criticalErrors ← criticalErrors + 1;
                break
            else
                point.x ← point.x + effectiveReviewers;
                point.y ← point.y + elapsedDays;
            end
        until simulationRuns < plannedSimulationRuns;
        point.x ← point.x/simulationRuns;         // averaging
        point.y ← point.y/simulationRuns;
        points ← points ∪ {point} ;
    end
    if criticalErrors > 0 then
        return criticalErrors * criticalFitness ;  // penalty for errors in strategy
    else
        return the area under the curve defined by points (points are interpolated by lines; area is
        calculated for x ∈ [minEffective, maxEffective]; if the range of points is smaller, it is assumed
        that the y value of the missing points is equal to the y value of the nearest point in points);
    end
end
```

**Algorithm S1b:** The algorithm used to simulate the review process.

```
structure ReviewThread{
    integer duration ;                  // duration, in days, of this review thread
    boolean hasReview ;   // indicates whether a review was received during the execution of this
    thread
    integer offset ← elapsedDays ;      // number of days after which the thread was started
} ;
Function simulation(batchSize: integer, editorialStrategy: editorial strategy):
    𝕋 ← { ReviewThread t_i | i = 1, 2, ..., batchSize};     // generate initial review threads
    threadsNumber ← batchSize;                                // initial number of threads
    receivedReviews ← 0;
    elapsedDays ← 0;
    effectiveReviewers ← batchSize;                           // initial number of reviewers
    while receivedReviews < 2 do
        elapsedDays ← min{t_i.offset + t_i.duration : t_i ∈ 𝕋}; // find the smallest number of days after
        which at least one of the review threads ended
        foreach (t_i ∈ 𝕋 | t_i.offset + t_i.duration = elapsedDays) do
            if t_i.hasReview = true then
                receivedReviews ← receivedReviews + 1
            end
            𝕋 ← 𝕋 \ t_i;              // remove the finished thread from the list of threads
            threadsNumber ← threadsNumber − 1;
        end
        if receivedReviews < 2 then
            newThreadsNumber ← editorialStrategy(state parameters);    // the strategy proposes a
            number of new threads that should be started based on available information
            if newThreadsNumber < 0 or
            newThreadsNumber + threadsNumber > batchSize or
            (threadsNumber = 0 and newThreadsNumber = 0) then
                return (criticalError ← true);
            else
                𝕋 ← 𝕋 ∪ { ReviewThread t_i | i = 1, 2, ..., newThreadsNumber};   // create new review
                threads
                effectiveReviewers ← effectiveReviewers + newThreadsNumber;
                threadsNumber ← threadsNumber + newThreadsNumber;
            end
        end
    end
    return elapsedDays and effectiveReviewers;
end
```
