# Supplementary material for: Artificial intelligence in peer review: How can evolutionary computation support journal editors?
Source: PLoS One. 2017 Sep 20;12(9):e0184711. doi: 10.1371/journal.pone.0184711 (PMC5607159; doi:10.1371/journal.pone.0184711)

Figure S1. The first 224 nodes of an editorial strategy with both active (black connections) and inactive (grey connections) nodes visible. The color of a node corresponds to the function realised by the node: light blue - input, red - subtraction, green - division, blue - multiplication, pink - addition, yellow - modulo.

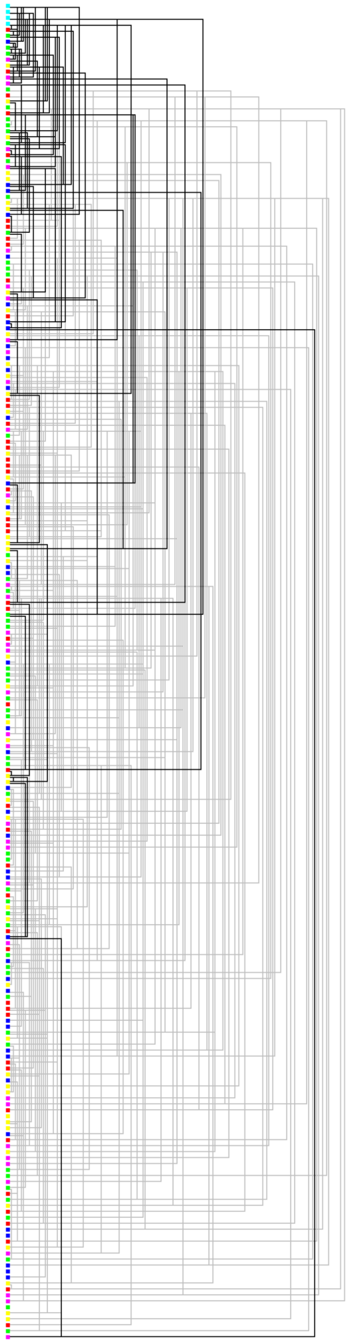

Supplement: S1 Fig — (PDF) [file pone.0184711.s004.pdf]
